# Continuous Filament Fabrication Technology and Its Mechanical Properties for Thin-Walled Component

**DOI:** 10.3390/ma19010144

**Published:** 2025-12-31

**Authors:** Tomasz Kozior, Jerzy Bochnia, Jiri Hajnys, Jakub Mesicek

**Affiliations:** 1 Faculty of Mechatronics and Mechanical Engineering, Kielce University of Technology, 25-314 Kielce, Poland; jbochnia@tu.kielce.pl; 2 Center of 3D Printing Protolab, Department of Machining, Assembly and Engineering Technology, Faculty of Mechanical Engineering, VSB Technical University Ostrava, 17. Listopadu 2172/15, 708 00 Ostrava, Czech Republic; jiri.hajnys@vsb.cz (J.H.); jakub.mesicek@vsb.cz (J.M.)

**Keywords:** thin-walled elements, CFF, MEX, mechanical properties, carbon fiber

## Abstract

The aim of the presented research is to assess the possibility of manufacturing thin-walled models using innovative 3D printing technology and to determine limitations. This article presents the results of tensile tests of the Continuous Filament Fabrication (CFF) technology for thin-walled sample models. Two types of materials were tested. The first material is pure ONYX based on polyamide, and the second is ONYX with an additional core made of carbon fiber. The paper presents the limitations of using the core in thin-walled structures, and for pure ONYX material, samples were made with different orientations on the 3D printer platform, which allowed determining the effect of the printing direction on the mechanical properties of the samples. In addition, microscopic photographs of the fracture of the broken samples were taken in the paper, based on which the defects of the technological process were identified. It was shown that the strength of thin-walled samples (1 mm, 1.4 mm, and 1.8 mm thick) printed in the Y direction is significantly greater than that of samples printed in the X and Z directions. For example, for 1 mm thick samples printed in the Y direction, the strength is 49.02 MPa, while for samples printed in the X and Z directions, it is 27.71 MPa and 21.28 MPa, respectively. The strength of samples (4 mm thick) reinforced with ONYX + OCF carbon fiber printed in the X direction is 191.36% greater than that of samples made of pure ONYX.

## 1. Introduction

Additive technologies, commonly referred to as 3D printing, are finding increasing application in various industrial sectors and has become an essential part in the Industry 4.0 [1,2]. Among the well-known technologies is material extrusion (MEX), also known as fused deposition modeling (FDM) or fused filament fabrication (FFF). The engineering materials from which parts are manufactured are crucial for industrial applications. These materials must withstand appropriate mechanical loads, be highly durable, and be economically attractive. In view of this, studies have attempted to mix a variety of materials to make composite filaments for MEX [3,4,5]. One such material is a polyamide filled with carbon microfibers, known as ONYX. ONYX can be modified with various fillers, including long carbon fibers, and is known as ONYX-CF (OCF). Due to its superior mechanical properties compared to other polymer materials used in 3D printing, this material has attracted interest from both industry and academia. Therefore, numerous studies, especially in recent years, have been devoted to research on this material. The largest group of articles describe the results of tests on the mechanical properties of ONYX, for example [6,7,8,9,10,11,12,13,14,15,16,17,18,19].

Extensive results of research on the mechanical properties of ONYX material reinforced with carbon, glass and Kevlar fibers are described in the paper [13]. Static and dynamic tests were performed. It was found that the fiber-reinforced samples exhibited tensile strengths 21, 17, and 15 times greater than those of pure ONYX, respectively. However, pure ONYX exhibited the highest ultimate strain, exceeding the fiber-filled samples by more than nine times. This paper also describes in detail the fatigue test results, particularly the effect of strain rate on strength and the suitability of individual materials for the construction of specific drone components.

The paper [16] presents an analysis of the three-point bending strength of a composite material consisting of polyamide doped with chopped carbon fiber and polyamide reinforced with continuous carbon fiber. The samples were produced by the material MEX in the X and Z printing directions. The test results showed a beneficial effect of continuous carbon fiber reinforcement on both the stiffness and strength of the material. The bending strength of the unreinforced composite printed in the X and Z directions was 77.34 MPa and 128.10 MPa, respectively, while the composite reinforced with continuous carbon fiber printed in the X and Z directions was 147.03 MPa and 143.38 MPa, respectively, which is 190% and 112% times greater than the bending strength of the samples without carbon fiber reinforcement, respectively.

The continuous carbon fibers and ONYX-FR matrix materials were used to build honeycomb-shaped cellular structures, which were then subjected to mechanical property tests [9]. ONYX-FR is the commercial name for flame-retardant polyamide materials filled with carbon fibers, manufactured using Markforged 3D printers (Markforged, Waltham, MA, USA). Based on tests, the structure with a 3.2 mm surface layer thickness and a 12.7 mm core size demonstrated the highest energy absorption and prevented delamination.

Interesting research results were presented in the paper [14]. The mechanical properties of 3D-printed composites consisting of ONYX and glass fibers obtained using Mark Two (Gen 2) 3D printing technology (Markforged, Waltham, MA, USA) are described. Optimal tensile strength was achieved with six face layers and a 45° honeycomb infill, leading to a 132.4% increase in tensile strength and a 24.2% improvement in hardness compared to a sample without fiber reinforcement.

In addition to articles on mechanical properties, there are also works on improving the geometric structure of the surface [18]. Specifically, 100 W laser source was used to reduce the surface roughness of 3D-printed ONYX parts. The surface finishing mechanism, the effect of laser process parameters (laser power, pulse frequency, and laser scanning path) on surface morphology, and the effect of the scanning path on the dimensional accuracy of the tested 3D-printed ONYX samples were determined. A significant surface roughness reduction of 91.15% was achieved on the S3 ONYX sample after polishing with a laser power of 50 W and a frequency of 50 kHz.

It is worth mentioning here a review article on MEX technology and the use of metal fillers to produce parts that, after appropriate thermal treatment, can replace more expensive technologies of sintering or laser melting of metal powders [20]. MEX polymer-metal composite technology is particularly useful in low-volume production, where total cost and production time are crucial. This article also characterizes the printing process parameters and their impact on the functional properties of the resulting components. It also discusses the process’s drawbacks, identifies gaps in existing research, and presents proposals for improving the technology.

From another perspective, OCF structures have been proven to effectively improve the flexural strength of thin-walled profiles of 3 mm [21]. In the study, the failure mechanisms of fibers and the ONYX matrix were characterized through both numerical simulations and microstructural investigations. Furthermore, the OCF system was employed to produce an off-axis tube with a thickness of 3.5 mm [22]. The tube was subjected to buckling tests, and it was reported that the addition of CF significantly strengthened the elbow sections. Microscopic observations at the elbows revealed not only how the tube failed under compressive forces but also confirmed that the fabrication process was geometrically accurate and repeatable, with the CF integrated seamlessly into the ONYX matrix.

Inspired by the above studies, this article investigates the static tensile strength of thin-walled components printed from ONYX material and OCF. Specifically, the tensile strengths of thin-walled samples with thicknesses of 1 mm, 1.4 mm, and 1.8 mm are compared with 4 mm thick reference samples of OCF. In addition, microscopic images of the fractures are analyzed to investigate the failure mechanisms of the components. The aim of this research is to assess the feasibility of manufacturing thin-walled components from ONYX and CF, evaluate their tensile strength and characterize their failure under tensile loading.

The primary limitation of this study, which allows for comparative analysis, is the sample thickness and the lack of international standards ISO/ASTM dedicated to thin-walled elements manufactured using 3D printing, which would standardize the research.

To sum up the current state of knowledge and based on the research experience of the authors of this publication, it can be stated that the issues of manufacturing thin-walled models by 3D printing by material manufacturers are not taken into account, and the research conducted in this area is fragmentary and requires supplementation, which is partially introduced in this publication. The novelty of this publication lies in the fact that the authors have never before encountered research results on thin-walled models produced using CFF technology. This makes this pioneering research, constituting, in a sense, the initial stage of research planned for the author of this article.

## 2. Materials and Methods

### 2.1. Materials

The composite tensile samples used in this study were prepared with Markforged^®^ Mark X7 FDM printer with a build volume of 330 × 270 × 200 mm^3^. The thickness can be set to between 50 and 250 µm. The OCF material (Waltham, MA, USA) is composed of ONYX and long CF. ONYX (Waltham, MA, USA) is nylon PA6 reinforced with short carbon fiber and can be further reinforced with long CF for further strength enhancement (see Table 1 for material properties). In this study, effect of thin wall thicknesses on samples printed using ONYX and OCF was investigated.

### 2.2. Methods

#### 2.2.1. CFF Technology

**Figure 1:**
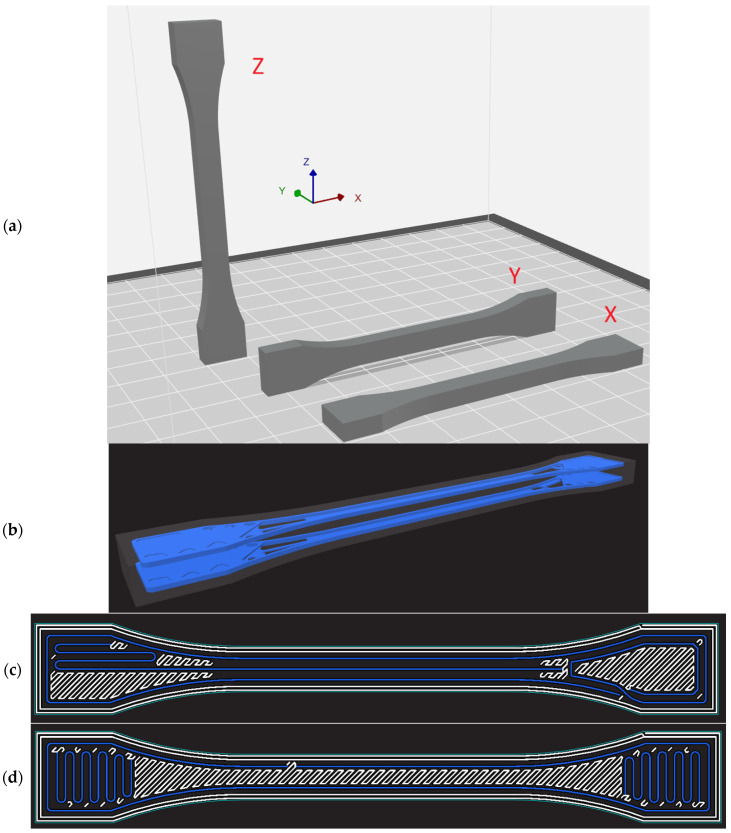
(**a**) Orientation of sample models on the build platform, (**b**) Isometric view, (**c**) layer 5–8, and (**d**) layer 25–28.

The tensile samples were designed following standard ISO 527-1:2019 [25], with varied thicknesses of 1 mm, 1.4 mm, 1.8 mm, and 4 mm. The example orientation of the OCF and ONYX samples are shown in Figure 1a, while the CF distribution is shown in Figure 1b–d.

It should be noted that due to the technological constraints, only OCF samples of 4 mm were printed only in X direction. Specifically, the CF was distributed on layer 5 to 8 and layer 25 to 28 of total 32 layers. These thickness of the sample and the positions of the CF layers were selected to satisfy the minimum geometric requirements of the CFF process, which mandates at least two solid ONYX layers above and below each reinforced region to ensure proper fiber anchoring and prevent delamination. Locating the reinforcement zones away from the outer surfaces also improves consolidation between the matrix and the continuous fibers. Furthermore, the spacing between both reinforcement zones provides a more uniform stiffness distribution across the specimen thickness.

Support structures were necessary only for samples printed in the Y orientation due to overhanging geometries. Continuous carbon fiber was used exclusively in the OCF-4-X specimen to reinforce selected layers of the ONYX matrix using Markforged’s Continuous Fiber Fabrication (CFF) technology. The specimens were prepared with thicknesses of 1.0 mm, 1.4 mm, 1.8 mm, and 4.0 mm were prepared in Eiger (https://markforged.com/software, accessed on 1 December 2025) (Markforged slicer, Waltham, MA, USA) to assess the effect of thin-wall geometries.

The process parameters can be found below in Table 2.

#### 2.2.2. Tensile Test

The static tensile test was performed using an Ispekt mini tensile testing machine (Hegewald & Peschke MPT GmbH, Nossen, Germany) with a 3 kN range. The Labmaster software (Version 2.5.3.21) (Hegewald & Peschke MPT GmbH, Nossen, Germany) included with the Inspekt mini machine set the test speed to 1 mm/min.

#### 2.2.3. Microscopy Analysis

Microscopic photographs were taken using two devices: the SMZ-140 microscope (MOTIC Company, Barcelona, Spain) and the MAGUS 650 (Levenhuk, Inc., Tampa, FL, USA).

#### 2.2.4. Dimensional Measurement

Sample dimensions were measured using an electronic micrometer with a measurement accuracy of 0.01 mm. Measurements were taken at three locations on the sample measurement database. Table 3 and Table 4 provide their mean values (*ā* and $\bar{b}$).

## 3. Results and Discussion

Below, in Section 3.1, Section 3.2 and Section 3.3, the results of geometry measurements, uniaxial tensile tests and microscopic photographs of the samples after breaking are presented.

### 3.1. Dimensional Accuracy

Table 3 and Table 4 present the results of sample geometry measurements taken using micrometer and caliper—manual measuring instruments with an accuracy of 0.01 mm. These measurement results were used during strength tests, where the average values of sample thickness and width were used in the Labmaster software to determine tensile strength. Table 3 and Table 4 also provide the mean values for each measurement series and the standard deviation (SD).

### 3.2. Tensile Test Results

**Figure 2:**
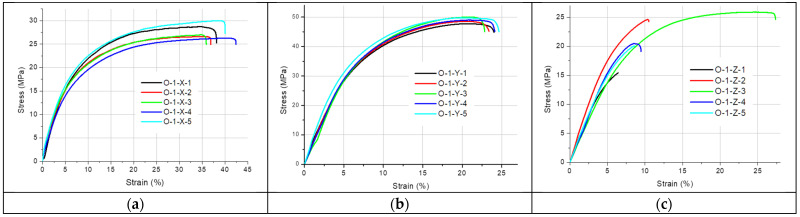
Stress–strain curves for the 1 mm thick specimens printed with ONYX material in: (**a**) the X orientation, (**b**) the Y orientation and (**c**) the Z orientation.

**Figure 3:**
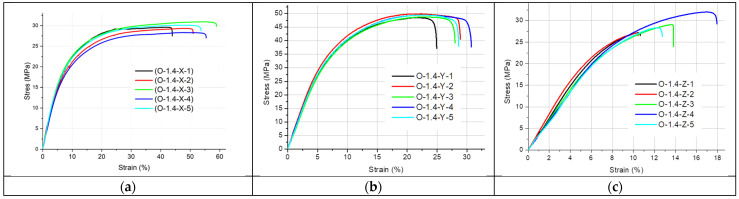
Stress–strain curves for the 1.4 mm thick specimens printed with ONYX material in: (**a**) the X orientation, (**b**) the Y orientation and (**c**) the Z orientation.

**Figure 4:**
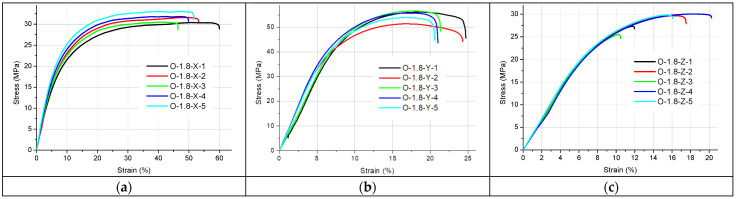
Stress–strain curves for the 1.8 mm thick specimens printed with ONYX material in: (**a**) the X orientation, (**b**) the Y orientation and (**c**) the Z orientation.

**Figure 5:**
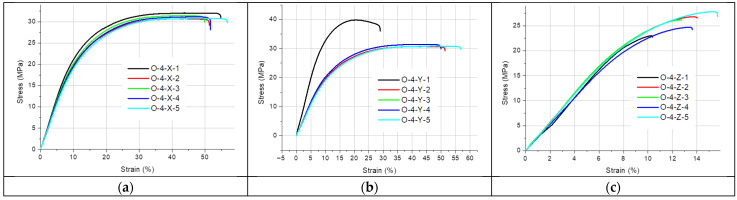
Stress–strain curves for the 4 mm thick specimens printed with ONYX material in: (**a**) the X orientation, (**b**) the Y orientation and (**c**) the Z orientation.

**Figure 6:**
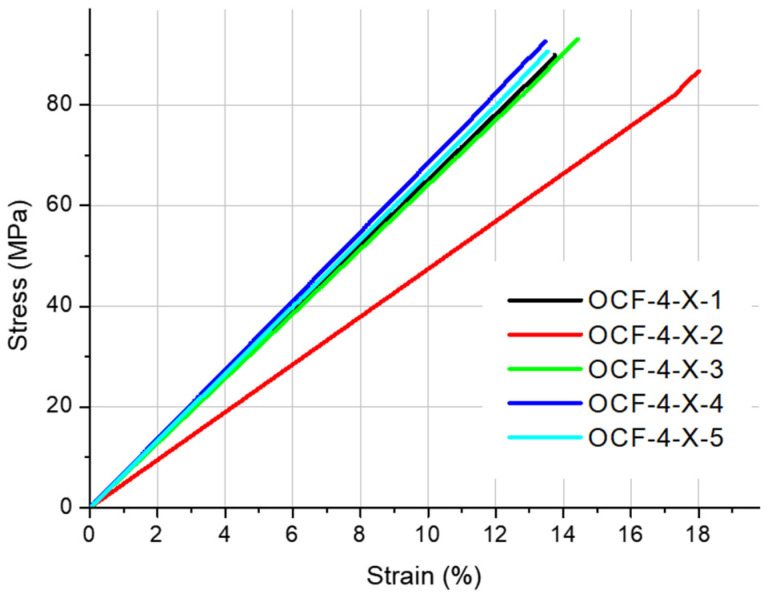
Stress–strain curves for the 4 mm thick specimens printed with ONYX material in X orientation.

Figure 2, Figure 3, Figure 4, Figure 5 and Figure 6 present the results of the static tensile test. Figure 2, Figure 3 and Figure 4 contain the test results for thin-walled models made only of ONYX material, and Figure 5 presents the results for a 4 mm thick reference sample also made of ONYX material. Furthermore, Figure 6 presents the results of tensile tests for 4 mm thick samples made in the Z direction using two materials: ONYX as the matrix and carbon fiber as the core. Table 5 and Table 6 present the results of tensile strength tests with the determination of the mean value $\bar{x}$, standard deviation *SD* and repeatability coefficient *V* (%) as the ratio of the standard deviation to the mean value.

Two basic problems can be encountered when testing the mechanical properties of samples produced using 3D printing technology, especially thin-walled samples:

- The sample dimensions do not match the dimensions of the CAD model,
- The tensile strength and strain at break, as well as other mechanical property indices, depend on the printing direction, i.e., orientation on the build platform (anisotropy).

Due to the aforementioned problems, shape deviations should be taken into account when analyzing test results and compared to the dimensions of the 3D CAD model. Furthermore, differences in mechanical properties should be analyzed depending on the printing direction (compared to the manufacturer’s specifications), as these are different for each 3D printing technology and printer used. To assess the thickness and width of the samples, the relative percentage differences in the average thickness Δ*a* and width Δ*b* were used, calculated using Formulas (1) and (2) [26]:

(1) $$\Delta a_{X,Y,Z}=\frac{\left|a-{\bar{a}}_{X,Y,Z}\right|}{a}\cdot 100\%,$$

where *a*—sample thickness according to CAD, e.g., *a* = 1.8 mm, ${\bar{a}}_{X,Y,Z}$—average sample thickness in a given measurement series based on Table 2 or Table 3. For example, for a thickness of *a* = 1.8 mm (Table 2) and orientation *X*, the value ${\bar{a}}_{X}$ = 1.94 mm.

(2) $$\Delta b_{X,Y,Z}=\frac{\left|b-{\bar{b}}_{X,Y,Z}\right|}{b}\cdot 100\%,$$

where *b*—sample width according to CAD, e.g., *b* = 5 mm, ${\bar{b}}_{X,Y,Z}$—average sample width in a given measurement series based on Table 2 or Table 3. For example, for width *b* = 5 mm and orientation *X* the value ${\bar{b}}_{X}$ = 5.16 mm.

**Figure 7:**
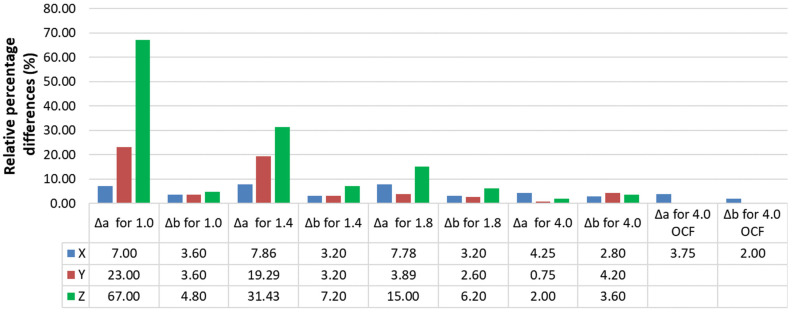
Relative percentage differences in thickness and width of samples made of ONYX and ONYX + OCF materials.

The calculated values of relative percentage differences in thickness Δ*a* and width Δ*b* for individual measurement series, calculated on the basis of data from Table 1 and Table 2, are shown in Figure 7. This is a column chart extended with a data table placed under the horizontal axis.

The largest relative percentage difference between the CAD dimensions of the designed 1 mm thick samples and the thickness of the ONYX samples printed in the Z orientation was 67%. Other significant differences were 31.43% for the series of samples with a designed thickness of 1.4 mm printed in the Z orientation and 19.29% and 23%, respectively, for the series of 1.4 mm and 1 mm samples printed in the Y orientation. The largest differences between the designed dimensions and the actual dimensions occur for thin-walled samples. For the reference 4 mm thick samples, such large differences were not observed. It should be added that sample thickness “a” always has larger deviations than width “b”, as shown in Figure 7 and Table 2.

**Figure 8:**
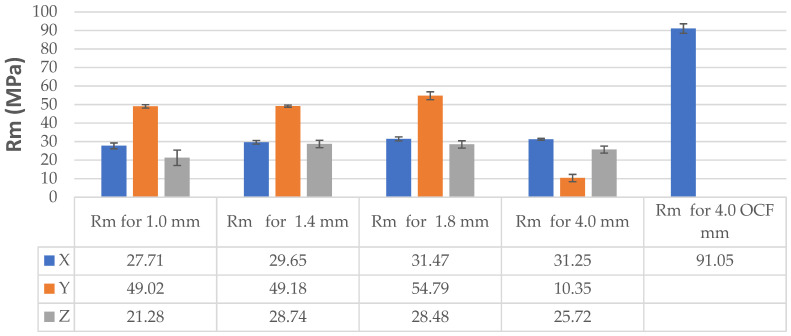
Tensile strength Rm for individual ONYX and OCF samples thicknesses and printing directions.

The tensile strength test results in Table 4 and Table 5 are presented visually in a column chart (Figure 8).

Analyzing the tensile strength of individual sample series, it can be concluded that the highest strength was achieved by samples printed in the X direction, made of OCF. This is evidenced by the tensile strength test results presented in Table 3 and visually presented in the bar graph (Figure 8). Comparison of 4 mm thick samples printed in the X direction shows that the strength of the OCF carbon fiber-reinforced samples is 191.36% greater than that of pure ONYX.

A certain pattern can be observed for the thin-walled samples. The strength of thin-walled samples printed in the Y direction was significantly higher than that of samples printed in the X and Z directions, ranging from 49.02 MPa to 54.79 MPa, while the X-direction printed samples had tensile strengths ranging from 27.71 MPa to 31.47 MPa. In contrast, the samples printed in the Z direction had a tensile strength ranging from 21.28 MPa to 28.48 MPa. No sudden rupture (without plastic deformation) was observed (see Figure 5) for the samples printed from ONYX material in the Z direction, as is the case with most 3D printing materials and technologies. The OCF sample (Figure 6) did not exhibit plastic deformation, achieving a high tensile strength. The test curves shown in Figure 6 have a completely different course compared to the curves in Figure 5 and determine completely different properties of the models produced with this technology. Furthermore, ONYX exhibits better plastic properties in all directions than OCF and other materials in the MEX technology, such as PLA and those enriched with carbon fibers, which very often exhibit no plastic deformation in the Z direction, as shown in the paper [27]. The tensile strength of the tested thin-walled samples is good in the group of polymeric materials, but it must be remembered that it is many times lower than that of similar elements manufactured by laser additive technology from metal powders [28]. Due to the large results distribution depending on the printing direction, the statistical calculations were verified and compared with the publication [29], which also analyzed models produced by 3D printing and where the author comprehensively presented the possibility of such an analysis.

### 3.3. Microscopic Analysis

**Figure 9:**
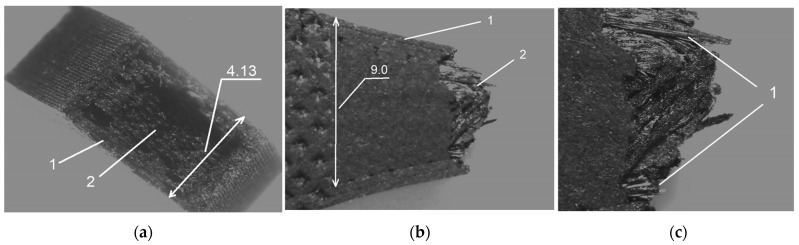
Cross-section of a broken OCF sample, (**a**,**b**)—6× magnification, 1—layer of pure ONYX material, 2—carbon fibers, (**c**)—12.5× magnification, 1—protruding carbon fibers.

**Figure 10:**
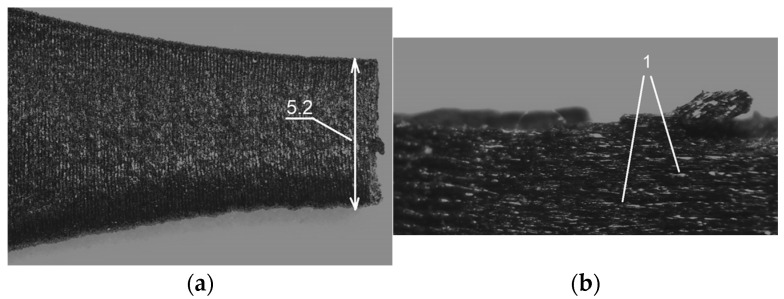
Cross-section of a torn sample of ONYX material with a thickness of 1.8 mm, (**a**)—6× magnification, (**b**)—12.5× magnification, 1—visible boundaries of the layers of the applied material.

Figure 9 and Figure 10 show photos of selected samples at the fracture site, taken with a stereoscopic microscope. Figure 9a–c show the cross-section of the fractured OCF sample.

Figure 9c, the sample after fracture, shows protruding individual carbon fibers (typical for composite materials), and Figure 10b shows the visible boundaries of the layers of the applied material.

**Figure 11:**
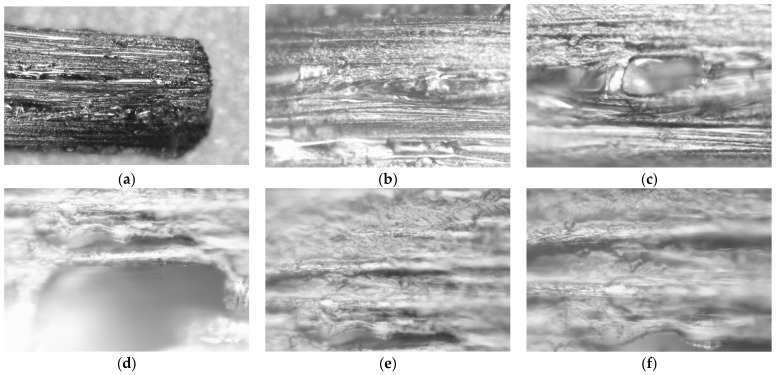
Carbon fiber bundle at the break point of the OCF sample shown in Figure 9c, (**a**)—100× magnification, (**b**)—200× magnification, (**c**)—400× magnification, visible material defect, (**d**)—1000× magnification, (**e**)—1000× magnification, (**f**)—1600× magnification.

Figure 11 shows microscopic images of the same carbon fiber bundle shown in Figure 9c. The images were taken with the Levenhuk Magus 650 microscope.

Carbon fibers embedded in the ONYX material form bundles that fill the interior of the printed objects, in this case, 4 mm thick samples. Such a bundle, detached from the cross-section of a cracked sample, is shown in Figure 11a. Then, by increasing the microscope magnification, a material defect (probably a gas cavity) was captured, as shown in Figure 11b–d. Such defects can negatively impact the mechanical properties of the manufactured elements. Figure 11e,f show the correct arrangement of carbon fibers in the ONYX material matrix.

## 4. Conclusions

The presented static tensile test results for thin-walled samples with thicknesses of 1 mm, 1.4 mm, and 1.8 mm compared to 4 mm thick reference samples made of ONYX material demonstrate that it is possible to produce thin-walled elements using 3D printing technology, as they are as strong as the 4 mm thick reference samples, and even exhibit higher strength in some print directions. The thin-walled samples printed in the Y direction have a strength of 49.02 MPa, 49.18 MPa and 54.79 MPa for samples with thicknesses of 1 mm, 1.4 mm and 1.8 mm, respectively, while the reference samples also printed in the Y direction have a strength of 10.35 MPa.

Comparison of 4 mm thick samples printed in the Z direction shows that the strength of the OCF carbon fiber-reinforced samples is 191.36% greater than that of pure ONYX.

The OCF samples did not exhibit plastic deformation, achieving high tensile strength. Samples made of ONYX material for all printing directions showed better plastic properties than OCF.

## Figures and Tables

**Table 1:** Properties of ONYX and CF in used [23,24].

Property	ONYX	CF
Tensile Strength (MPa)	40	800
Tensile Modulus (GPa)	2.4	60
Tensile Strain at Break (%)	25	1.5

**Table 2:** Printing process parameters.

Support material	ONYX (used only for Y-oriented samples)
Reinforcement material (OCF)	Continuous Carbon Fiber
Nozzle temperature (ONYX)	274 °C
Nozzle temperature (CF)	252 °C
Layer height (ONYX samples)	0.10 mm
Layer height (OCF sample)	0.125 mm
Infill type	Solid fill (100%)
Wall layers	2
Roof/floor layers	2
Continuous fiber layers (OCF)	4
Fiber layout (OCF)	Isotropic (0°/45°/90°/135°), 2 concentric rings
Fiber placement mode	Entire Group
Slicing mode	Cloud slicing ON

**Table 3:** Dimensions of samples made with MEX technology from ONYX material.

No.	*ā* (mm)	$\bar{b}$ (mm)	No.	*ā* (mm)	$\bar{b}$ (mm)	No.	*ā* (mm)	$\bar{b}$ (mm)	No.	*ā* (mm)	$\bar{b}$ (mm)
O-1-X-1	1.09	5.27	O-1_4-X-1	1.52	5.13	O-1_8-X-1	1.93	5.24	O-4-X-1	4.15	5.09
O-1-X-2	1.07	5.13	O-1_4-X-2	1.51	5.11	O-1_8-X-2	1.95	5.16	O-4-X-2	4.16	5.24
O-1-X-3	1.06	5.13	O-1_4-X-3	1.47	5.20	O-1_8-X-3	1.97	5.17	O-4-X-3	4.19	5.10
O-1-X-4	1.12	5.19	O-1_4-X-4	1.52	5.21	O-1_8-X-4	1.93	5.10	O-4-X-4	4.18	5.12
O-1-X-5	1.01	5.16	O-1_4-X-5	1.53	5.13	O-1_8-X-5	1.91	5.13	O-4-X-5	4.19	5.17
$\bar{x}$	1.07	5.18	$\bar{x}$	1.51	5.16	$\bar{x}$	1.94	5.16	$\bar{x}$	4.17	5.14
*SD*	0.04	0.06	*SD*	0.02	0.05	*SD*	0.02	0.05	*SD*	0.02	0.06
O-1-Y-1	1.27	5.21	O-1_4-Y-1	1.73	5.21	O-1_8-Y-1	1.88	5.13	O-4-Y-1	4.04	5.19
O-1-Y-2	1.28	5.20	O-1_4-Y-2	1.66	5.14	O-1_8-Y-2	1.85	5.13	O-4-Y-2	3.98	5.13
O-1-Y-3	1.19	5.19	O-1_4-Y-3	1.71	5.17	O-1_8-Y-3	1.87	5.15	O-4-Y-3	4.05	5.23
O-1-Y-4	1.29	5.17	O-1_4-Y-4	1.63	5.14	O-1_8-Y-4	1.89	5.12	O-4-Y-4	4.07	5.27
O-1-Y-5	1.14	5.14	O-1_4-Y-5	1.60	5.12	O-1_8-Y-5	1.86	5.12	O-4-Y-5	4.03	5.24
$\bar{x}$	1.23	5.18	$\bar{x}$	1.67	5.16	$\bar{x}$	1.87	5.13	$\bar{x}$	4.03	5.21
***SD***	0.07	0.03	***SD***	0.05	0.04	***SD***	0.02	0.01	***SD***	0.03	0.05
O-1-Z-1	1.84	5.41	O-1_4-Z-1	1.83	5.35	O-1_8-Z-1	2.05	5.29	O-4-Z-1	4.08	5.12
O-1-Z-2	1.54	5.24	O-1_4-Z-2	1.83	5.33	O-1_8-Z-2	2.07	5.31	O-4-Z-2	4.07	5.10
O-1-Z-3	1.83	4.91	O-1_4-Z-3	1.85	5.41	O-1_8-Z-3	2.07	5.32	O-4-Z-3	4.10	5.11
O-1-Z-4	1.57	5.33	O-1_4-Z-4	1.83	5.35	O-1_8-Z-4	2.08	5.35	O-4-Z-4	4.05	5.46
O-1-Z-5	1.56	5.29	O-1_4-Z-5	1.86	5.35	O-1_8-Z-5	2.07	5.29	O-4-Z-5	4.09	5.11
$\bar{x}$	1.67	5.24	$\bar{x}$	1.84	5.36	$\bar{x}$	2.07	5.31	$\bar{x}$	4.08	5.18
***SD***	0.15	0.19	***SD***	0.01	0.03	***SD***	0.01	0.02	***SD***	0.02	0.16

**Table 4:** Dimensions of samples made using MEX technology from OCF material.

No.	*ā* (mm)	$\bar{b}$ (mm)
OCF-4-X-1	4.13	5.08
OCF-4-X-2	4.17	5.07
OCF-4-X-3	4.15	5.13
OCF-4-X-4	4.15	5.11
OCF-4-X-5	4.14	5.09
$\bar{x}$	4.15	5.10
*SD*	0.015	0.024

**Table 5:** Ultimate tensile strength and the maximum elongation at failure at the point of breakage of samples made with MEX technology from ONYX material.

No.	*R_m_* (MPa)	*ε_m_* (%)	No.	*R_m_* (MPa)	*ε_m_* (%)	No.	*R_m_* (MPa)	*ε_m_* (%)	No.	*R_m_* (MPa)	*ε_m_* (%)
O-1-X-1	28.71	38.2	O-1_4-X-1	29.60	44.4	O-1_8-X-1	30.40	54.6	O-4-X-1	32.07	54.8
O-1-X-2	26.63	37.0	O-1_4-X-2	29.32	50.0	O-1_8-X-2	31.60	53.2	O-4-X-2	30.72	51.3
O-1-X-3	26.98	36.4	O-1_4-X-3	30.92	58.9	O-1_8-X-3	30.48	46.4	O-4-X-3	31.42	49.9
O-1-X-4	26.27	42.5	O-1_4-X-4	28.31	55.4	O-1_8-X-4	31.82	49.9	O-4-X-4	31.20	51.7
O-1-X-5	29.98	40.1	O-1_4-X-5	30.10	53.7	O-1_8-X-5	33.06	51.7	O-4-X-5	30.85	56.8
$\bar{x}$	27.71	38.8	$\bar{x}$	29.65	52.7	$\bar{x}$	31.47	51.2	$\bar{x}$	31.25	52.9
***SD***	1.58	2.5	***SD***	0.97	5.5	***SD***	1.09	3.2	***SD***	0.53	2.8
***V***	5.7	6.4	***V***	3.3	10.4	***V***	3.5	6.3	***V***	1.7	5.3
O-1-Y-1	47.75	24.3	O-1_4-Y-1	48.49	25.0	O-1_8-Y-1	56.15	24.7	O-4-Y-1	39.82	28.9
O-1-Y-2	48.58	23.7	O-1_4-Y-2	49.88	29.0	O-1_8-Y-2	51.43	24.3	O-4-Y-2	40.19	28.2
O-1-Y-3	50.02	23.1	O-1_4-Y-3	48.72	28.1	O-1_8-Y-3	56.58	21.3	O-4-Y-3	38.67	30.2
O-1-Y-4	48.99	24.5	O-1_4-Y-4	49.40	30.8	O-1_8-Y-4	55.78	21.0	O-4-Y-4	36.16	27.9
O-1-Y-5	49.78	24.9	O-1_4-Y-5	49.39	28.7	O-1_8-Y-5	54.00	20.6	O-4-Y-5	36.10	28.7
$\bar{x}$	49.02	24.1	$\bar{x}$	49.18	28.3	$\bar{x}$	54.79	22.4	$\bar{x}$	38.19	28.8
***SD***	0.92	0.7	***SD***	0.56	2.1	***SD***	2.12	2.0	***SD***	1.96	0.9
***V***	1.9	2.9	***V***	1.1	7.4	***V***	3.9	8.9	***V***	5.1	3.1
O-1-Z-1	15.42	6.4	O-1_4-Z-1	27.18	10.7	O-1_8-Z-1	27.34	12.0	O-4-Z-1	23.02	10.4
O-1-Z-2	24.61	10.5	O-1_4-Z-2	27.11	10.5	O-1_8-Z-2	29.67	17.5	O-4-Z-2	26.82	14.1
O-1-Z-3	25.88	27.4	O-1_4-Z-3	29.07	13.8	O-1_8-Z-3	25.54	10.5	O-4-Z-3	26.21	12.8
O-1-Z-4	20.47	9.8	O-1_4-Z-4	32.00	17.9	O-1_8-Z-4	30.04	20.3	O-4-Z-4	24.72	13.7
O-1-Z-5	20.04	8.6	O-1_4-Z-5	28.35	12.8	O-1_8-Z-5	29.83	16.1	O-4-Z-5	27.82	15.7
$\bar{x}$	21.28	12.6	$\bar{x}$	28.74	13.1	$\bar{x}$	28.48	15.3	$\bar{x}$	25.72	13.3
***SD***	4.15	8.4	***SD***	2.00	3.0	***SD***	1.98	4.0	***SD***	1.88	2.0
***V***	19.5	6.7	***V***	7	22.9	***V***	7	26.1	***V***	7.3	15

**Table 6:** Ultimate tensile strength and the maximum elongation at failure at the point of breakage of samples made with MEX technology from OCF material.

No.	*R_m_* (MPa)	*ε_m_* (%)
OCF-4-X-1	92.05	13.8
OCF-4-X-2	86.74	18.0
OCF-4-X-3	93.15	14.4
OCF-4-X-4	92.64	13.5
OCF-4-X-5	90.65	13.6
$\bar{x}$	91.05	14.7
***SD***	2.58	1.9
***V***	2.8	12.9

## Data Availability

The original contributions presented in this study are included in the article. Further inquiries can be directed to the corresponding author.
